# Comparison of psychosocial and emotional consequences of childhood strabismus on the families from rural and urban India

**DOI:** 10.4103/0301-4738.53053

**Published:** 2009

**Authors:** Mihir Kothari, Suwarna Balankhe, Rinkle Gawade, Svetlana Toshnival

**Affiliations:** 1 Mahatme Eye Hospital and Eye Bank, 16, Central Excise Colony, Chhatrapati Square, Wardha Road, Nagpur, India; 2 Jyotirmay Eye Clinic and Pediatric Low Vision Center, 205, Ganatra Estate, Pokhran Rd., No. 1, Khopat, Thane West – 400 601, India; 3 Aditya Jyot Eye Hospital, 153, Maj. Parmeshwaran Rd., No. 9, Wadala, Mumbai - 400 031, Maharashtra, India

**Keywords:** Children, quality of life, squint

## Abstract

**Purpose:**

To compare the psychosocial consequences of horizontal comitant strabismus in children between the families of urban and rural India.

**Materials and Methods:**

In this cohort study, an eight-question quality-of-life instrument was administered by trained staff to the guardians of strabismic children from rural and urban areas by a live interview.

**Results:**

This study included 93 strabismic-children aged 4-16 years of which 52 were females. Forty-one had esodeviation and 52 had exodeviation. Seventy per cent parents were extremely distressed due to squint, 65% were extremely distressed due to people's remarks, 65% were extremely worried, 55% children were extremely distressed due to people's remarks, 57% children were severely ostracized, 38% had severe difficulty in communication and 50% had difficulty to cope; 64% parents were not advised a corrective surgery. The difference between families from rural and urban areas, or whether a male child was affected or a female child or for an esodeviation or an exodeviation was statistically not significant. The questionnaire had a good internal consistency (Cronbach's Alpha = 0.71).

**Conclusions:**

There was a significant negative psychosocial and emotional impact of childhood strabismus that was not affected by the rural or urban location of the family or the gender of the strabismic child or type of the deviation. The quality-of-life instrument can be used as part of the clinical examination for strabismic children.

The primary goal of strabismus surgery is to align the visual axes to achieve binocular single vision. Other advantages of strabismus correction include improvement in abnormal head posture,[1–3] expansion of visual field,[4,5] restoration of stereo acuity,[6–8] centralization of visual field,[9] elimination of diplopia,[10,11] improvement of ocular motility,[12–14] improvement in the psychomotor development[15] and restoration of *normal* appearance.

Children and adults with strabismus often suffer from several psychosocial and emotional consequences viz. poor self image, negative social bias, ridicule at school, ostracization, depression, anger and outrage, increased social anxiety, poor interpersonal relationship, inhibition and poor job opportunities in adults.[16–27] It makes sense to study the quality of life of the strabismic patients and the beneficial effects of its correction and not call those surgeries as *cosmetic* when they are performed to improve the appearance. Several strabismologists have condemned the use of the term *cosmetic* in the treatment of strabismus in such situations.[28–30] By dictionary definition, cosmetic surgery is one that is performed to enhance or beautify. However, strabismus is a pathological state due to an underlying disease process, which is associated with abnormal binocular vision and leads to an objective deviation from the *normal* appearance that affects the quality of life.

In recent years clinicians have realized the importance of health-related quality of life (HRQL) studies. HRQL is important for measuring the impact of a chronic disease. Physiologic measures provide information to clinicians but are of limited interest to patients; they often correlate poorly with functional capacity and wellbeing, the areas in which patients are most interested and familiar. Another reason to measure HRQL is the commonly observed phenomenon that two patients with the same clinical criteria often have dramatically different responses. Some patients with strabismus may continue to live without significant psychosocial or emotional consequences while others may develop inhibition and major depression.

So far, there has been no study from India on the quality of life of the strabismic children and their families. The aim of this study was to assess and compare psychosocial and emotional consequences of childhood strabismus on the families and strabismic children from rural and urban India.

## Materials and Methods

This prospective, interventional study included a cohort of strabismic patients from rural area (Group 1) and urban area (Group 2). Patients in Group 1 were recruited from two widely separated districts of Maharashtra state. The children in Group 1 underwent state-sponsored free strabismus surgery under Sarva Shiksha Abhiyan in the month of January 2008 and April 2008. They underwent free eye surgeries on the basis of their low socioeconomic status, rural location of their residence and child studying in a state-run school. The education officers of their respective Zilla Parishads had identified and referred these children. The subjects in Group 2 were recruited from a private practice located in an urban area. Only children aged four to 16 years with manifest horizontal comitant strabismus measuring at least 15 prism diopter (PD) in primary position were included in this study. Children with neurological deficits, high ametropia, nystagmus, paralytic, restrictive or vertical squints, intermittent deviations, accommodative esotropia, chromosomal anomalies and other cosmetic deformities were excluded.

Two trained staff administered an eight-question quality-of-life instrument [Table 1] by live interviews in their native language. Both the interviewers were well-experienced and fluent in the native language. In the order of preference, the interviewee was the mother or the father or the guardian accompanying the child. The interviewer ensured that every question was understood well and answered.

**Table 1:** Quality-of-life questionnaire

How distressed do you get when you see (squint in the) face of your child?			
A. Not at all	B. A little	C. Moderately	D. Extremely
How distressed do you get when other people remark about the facial feature (squint) of your child?			
A. Not at all	B. A little	C. Moderately	D. Extremely
How much do you worry about the squint of your child?			
A. Not at all	B. A little	C. Moderately	D. Extremely
How distressed does the child get when other people remark about the facial feature (squint) of your child?			
A. Not at all	B. A little	C. Moderately	D. Extremely
How ostracized does the child get due to facial feature (squint)?			
A. Not at all	B. A little	C. Moderately	D. Extremely
How negatively does the facial feature (squint) affect the child's nonverbal communication?			
A. Not at all	B. A little	C. Moderately	D. Extremely
Has squint in your child affected your closeness with him/her?			
A. Yes	B. No		
Why did you not go for eye surgery for your child till now?			
A. No one advised	B. Afraid of complications	C. No access to healthcare	D. Financial constraints

The procedures followed were in accordance with the ethical standards as stated in the Helsinki Declaration of 1975 and revised in 2000.

The data was entered in a Microsoft Excel sheet and analyzed using NCSS (Number cruncher statistical software, 2007, Kaysville, Utah, USA). The internal consistency of the questionnaire was determined with Cronbach's Alpha. Two-tailed *t* test with equal variance was used for the comparison of continuous variables and Chi Square test was used for the comparison of discrete variables. *P* value <0.05 was considered significant.

## Results

A total of 93 patients were included. Their distribution and demographics are shown in Table 2. The distribution of the responses of the interviewees for items 1 to 6 from Group 1 and Group 2 are mentioned in Table 3. The differences in responses were statistically not significant between the two groups. Cronbach's Alpha for items 1 to 6 was 0.71.

**Table 2:** Distribution and demographic characteristics of patients in group 1 and group 2

	Group 1	Group 2	*P* value
Number of patients	77	16	
Mean age (years) ± SD (Range)	9 ± 2.7 (4-16)	10 ± 3.9 (4-16)	0.17 (*t* test)
M:F	30:47	11:5	0.06 (Chi square)
Eso:Exo	33:44	8:8	0.8 (Chi square)
Mean deviation ± SD (Range)	43.1 Δ ± 15 (15-85)	48.2 Δ ± 18.7 (16-90)	0.24 (*t* test)

**Table 3:** Percentage and count distribution of item response analysis

Variable	Gp 1 % (n)				Gp 2 % (n)			
		
	A	B	C	D	A	B	C	D
Q1	5.2(4)	3.9(3)	20.8(16)	70.1(54)	6.3(1)	25(4)	0(0)	68.8(11)
Q2	16.9(13)	1.3(1)	15.6(12)	66.2(51)	31.3(5)	6.3(1)	12.5(2)	50(8)
Q3	11.7(9)	3.9(3)	20.8(16)	63.6(49)	12.5(2)	6.3(1)	0(0)	81.3(13)
Q4	19.5(15)	5.2(4)	18.1(14)	57.1(44)	37.5(6)	12.5(2)	12.5(2)	37.5(6)
Q5	6.5(5)	14.3(11)	22.1(17)	57.1(44)	31.3(5)	12.5(2)	6.25(1)	50(8)
Q6	13(10)	22.1(17)	27.3(21)	37.7(29)	12.5(2)	18.8(3)	18.75(3)	50(8)

Gp: Group, Q: Question

In none of the cases, the presence of squint in the child had affected the proximity of the parents with the child.

**Figure 1:**
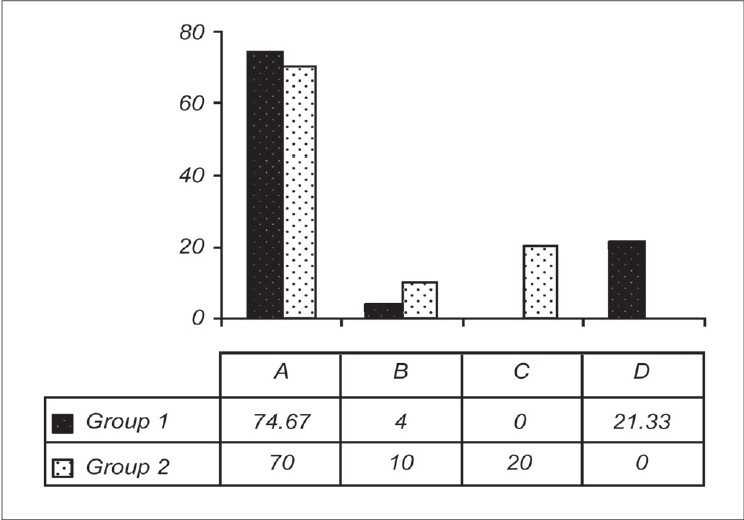
Item response analysis graph showing distribution of responses (in percentage) to question 8. The item description is plotted on the × axis (the possible responses are denoted as A/B/C/D) and the frequency is plotted on the y axis. Responses of the individuals from group 1 are marked in dotted black boxes and group 2 are marked in dotted white boxes

Fig. 1 shows comparative distribution of the parent's response to Question 8. The majority of the families were not advised a squint surgery irrespective of whether the family consulted a physician (pediatrician/ophthalmologist) in an urban or a rural area. The responses of the families when a male child was affected with squint were statistically not different compared to the families in which a female child had a squint, Also the statistical difference was not significant for the esodeviations compared to the exodeviations. However, larger angle (>40 PD) was a cause for more worries (*P* = 0.04).

## Discussion

In this study we found that the children and parents suffer from significant negative psychosocial and emotional consequences of strabismus. Our results are very similar to the results of the previous investigators.[16,17,22,25,31,32] The psychosocial and emotional effects on the children with strabismus are many. It is not difficult to imagine a child with strabismus to get noticed and ridiculed by the peers at school or at home. The age of emergence of negative attitudes towards strabismus in children is early.[31] Most children recognize and develop a dislike for strabismus by four years of age.[31] They begin to ridicule or tease a child who suffers from strabismus, resulting in isolation (ostracization) or hostility of the strabismic child. Reports from developmental psychologists reveal that a child develops mirror recognition of the self between two to four years of age.[32,33] These children can appreciate an abnormality in their appearance and if they suffer from any handicap or cosmetic deformity at this age, multiple psychosocial and emotional changes may affect them negatively.[34] Hence we believe that the right age for intervention to correct the strabismus should be before four years of age. Also, by this age a child starts interacting with other children of the same age and has to work under peer pressures.

In the present study, most parents reported ostracization and reduced communication skills of their child as compared to their peers. Few parents reported about the hostility of their child when remarked to have *squint eyes*. Certainly these emotional consequences need to be addressed. Investigators and clinicians may include questions pertinent to the assessment of such emotional behavior in strabismic children in their future work.

It is known that having a child whose appearance is distorted can cause trouble undertaking the motherhood role.[35–37] Mothers who have children with strabismus suffer from higher depression score, sense of failure, sense of guilt, pessimism and psychological complaints.[35] It also adversely affects their family relationships and proximity to the child. Most singularly, parents in this study vehemently denied reduced proximity with their child. This may be the result of the differences in the cultural principles of the Indian society. The clinicians and investigators may not include this item in the future questionnaire.

Contrary to our belief and a previous study on adult patients[38] with squint where exodeviation and male gender were reported to have lesser negative impact of strabismus, in this study we did not find a significant difference in responses for an exodeviation compared to an esodeviation or whether it was a male child or a female child that was affected with the squint.

The families in our rural group included parents working in the agricultural industry (most of them daily laborers) and their socioeconomic status and literacy status was far inferior to that of the parents from urban India. With comparison between rural and urban location, apart from the socioeconomic status, literacy and occupation of the parents, cultural differences also get simultaneously evaluated. In this study there was no significant difference in the responses of the families from urban India compared to that from rural India. This indicates that the negative impact of the strabismus on the child is experienced nearly equally in both the extreme strata of the community.

In this study worse responses were found for deviation > 40 PD compared to deviations lesser than 40 PD suggesting some relationship with the degree of deviation.

The last item in this questionnaire was included to obtain information on causes of delayed surgery in the two groups. It appears that there is still a lot of unawareness among the medical caregivers and lay public about the optimal time for surgical correction of strabismus in children. Pertinent measures are needed to disseminate this information.

In this study we have used parental proxies to assess the functional effect of strabismus in children. Although, the parental proxies were reported to provide meaningful indicators to assess the psychosocial impact of strabismus in children,[16] the clinician must be cautioned that proxy reports of more observable domains, such as physical functioning and cognition, are more correlated with reports from the patients themselves. For functional limitations, proxy respondents tend to consider patients more impaired (they overestimate patient dysfunction relative to the patients themselves). This is particularly characteristic of those proxies with the greatest contact with the respondent.[39]

Although we could not evaluate the effect of surgical correction, a previous study by Archer *et al.*,[16] has demonstrated that statistically significant improvements can be seen in the social, emotional, and functional measures of the children's health status after surgical realignment. This indicates significant psychosocial benefits afforded by strabismus surgery to improve the quality of life of children with strabismus.[16] Calling these surgeries cosmetic demeans the benefits the children and families gain with the squint correction. By fixing their deformities, we positively change the way others interact, react and relate with them, helping shape how well they learn, socialize and adapt to the world around them.

Further studies are required with a larger sample size and a design to evaluate the effect of surgical correction on the quality of life of the strabismic children. Comparison of the responses from different ethnic groups from different states of India may reveal differences in the HRQL of strabismic children or lack there of.

In summary there was a significant negative psychosocial impact of strabismus on parents and children with strabismus. This phenomenon was universal and was not affected by the rural or urban location of the family, gender of the child who had the strabismus or type of the deviation. The quality-of-life instrument used by us had good internal validity and can be used as part of the clinical examination for strabismic children. Further studies to evaluate improvement in the quality of life after a successful squint surgery are required.
